# A Case of Acquired Haemophilia A in a Patient with Chronic Myelomonocytic Leukaemia

**DOI:** 10.1155/2019/8612031

**Published:** 2019-02-27

**Authors:** Takeshi Araki, Shinya Ohata, Kohei Okamoto, Kazuhide Morimoto, Mana Hiraishi, Shinichiro Yamada, Ishikazu Mizuno, Takeshi Sugimoto

**Affiliations:** ^1^Department of Hematology and Oncology, Kita-Harima Medical Center, Hyogo, Japan; ^2^Department of Nephrology, Kobe University Hospital, Hyogo, Japan; ^3^Division of Laboratory Medicine, Kita-Harima Medical Center, Hyogo, Japan; ^4^Department of Cardiology, Kita-Harima Medical Centre, Hyogo, Japan; ^5^Department of Hematology, Hyogo Cancer Center, Akashi, Japan

## Abstract

A 67-year-old male, with a known diagnosis of myelodysplastic syndromes with multilineage dysplasia (MDS-MLD) was admitted to our hospital with a primary complaint of subcutaneous bleeding in his left thigh. Laboratory data showed anaemia and prolongation of activated partial thromboplastin time (85.8 s, normal range 24–39 s) without thrombocytopenia. Coagulation factor VIII (FVIII) activity was less than 1% (normal range 60–150%), and a FVIII inhibitor was identified and quantified at 166 BU/mL to indicate a diagnosis of acquired haemophilia A (AHA). A recent, but sustained circulating monocytosis (>1 × 10^9^/L) was observed, which combined with elevated numbers of neutrophil and monocytic cells in the marrow, suggested evolution of MDS-MLD to chronic myelomonocytic leukaemia (CMML), coinciding with AHA. Further analysis revealed a karyotype of 46, XY, *i*(14) (q10), which was the same abnormality previously identified in the patient. To treat bleeding caused by AHA, steroid and activated prothrombin complex concentrate were administered. Azacitidine (AZA) was used to treat CMML. During the clinical course, bleeding partially improved; however, subsequent acute myocardial infarction occurred on day 87. Worsening bone marrow failure was observed 4 months after the original admission, despite administration of AZA therapy, and the patient died due to bleeding from AHA. This case suggests that the evolution of MDS to CMML status can be associated with AHA conferring a bleeding tendency.

## 1. Introduction

Acquired haemophilia A (AHA) is a bleeding disorder caused by antibodies against coagulation factor VIII (FVIII). The reported incidence of AHA is between 1.0 and 1.5 cases per million population per year [1, 2]. The resulting coagulopathy can be life threatening, and if left untreated, has an overall mortality rate of 7.9%–22% [3]. Almost half of the AHA cases presented are idiopathic; the other half are associated with autoimmune disease, malignancy, dermatological disease, or pregnancy [4, 5]. The case presented herein had AHA with chronic myelomonocytic leukaemia (CMML).

## 2. Case Presentation

**Figure 1:**
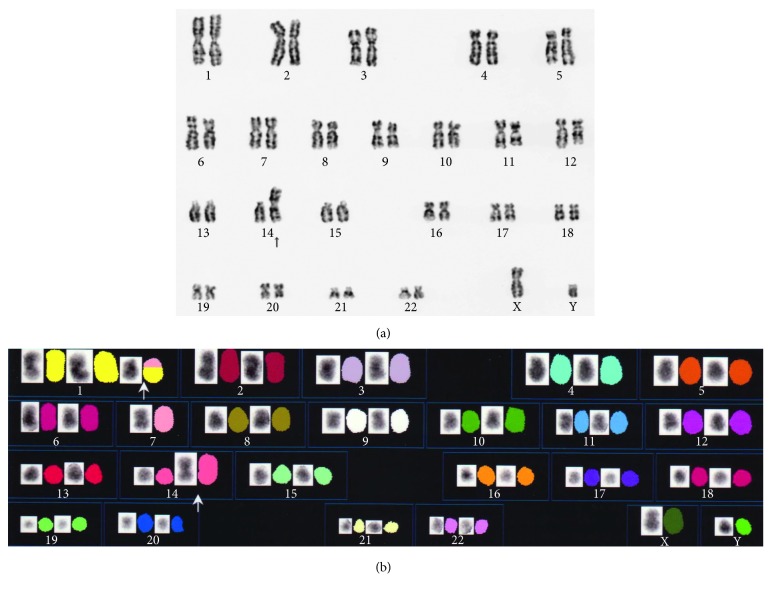
(a) G-banding karyotype showing the *i*(14q) (q10) with a bone marrow aspiration test at two years before admission. (b) Karyotype analysis using spectral karyotyping (SKY) method with a bone marrow aspiration test on day 138. SKY analysis identified an anomaly [idic (14), +1, and der (1; 7) (q10; p10)], which was the same abnormality as the result by G-banding analysis (data not shown).

A 67-year-old man presented to our hospital with a primary complaint of subcutaneous bleeding in his left thigh and development of purpura, over the last 3 months. It had been pointed out to him that there was a mild anaemia with haemoglobin level of 10.4 g/dL. He had been diagnosed two years previously with myelodysplastic syndromes with multilineage dysplasia (MDS-MLD) considered in the context of the 2016 World Health Organization (WHO) classification criteria. The 46, XY, *i*(14) (q10) abnormality was detected in 100% of marrow metaphases (Figure 1(a)). The bone marrow blast was 3.3%. He was categorized as low risk in Revised International Prognostic Scoring System (IPSS-R), and he had not received cytokine therapy or blood transfusion. He had a medical history of hypertension, type 2 diabetes mellitus, spinal canal stenosis, and idiopathic osteonecrosis of the femoral head and underwent right total hip arthroplasty (r-THA).

**Figure 2:**
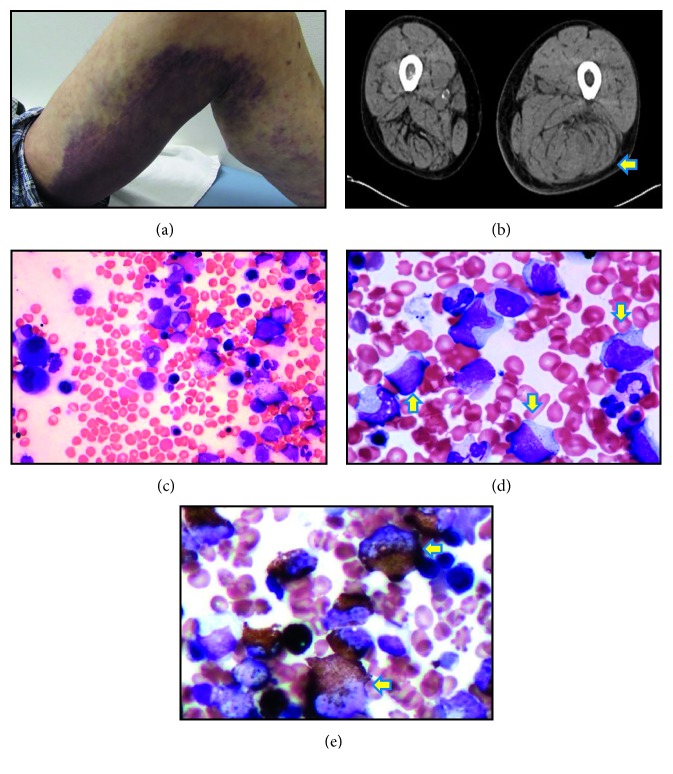
(a) Swelling of the left thigh with subcutaneous hemorrhage. (b) Computed tomography showing a large muscle bleed in the left quadriceps femoris muscle (arrow). (c–e) Bone marrow aspiration test on hospital day 24 (HE staining). (c) Normocellular marrow with an adequate proportion of erythrocytes (×400). (d) Granular cells possess a vacuole; 12% of nucleated cell count (NCC) were classified as monocytic cells (×1000, arrow). (e) Abnormal cells stained positive for peroxidase staining (×1000, arrow).

Physical examination showed conjunctival pallor and a swelling on the left thigh with overlying bruising (Figure 2(a)). Computed tomography revealed a large bleed in the left quadriceps femoris muscle (Figure 2(b)). Laboratory data showed the following: white blood cell count, 13.6 × 10^9^/L (normal range 3.5–9.7 × 10^9^/L); monocyte count, 4.6 × 10^9^/L (monocyte percentage, 33.6%); haemoglobin level, 8.8 g/dL (13.2–17.2 g/dL); haematocrit, 28.1% (40–52%); and platelet count, 258 × 10^9^/L (140–370 × 10^9^/L) (Table 1). On review, the monocyte count on three months previously too had been elevated (3.0 × 10^9^/L). Activated partial thromboplastin time (APTT) was significantly prolonged (85.8 s, normal range 24–39 s). Prothrombin time was within the normal range (10.5–14 s). Mixing studies demonstrated that the prolongation of APTT was not corrected by the addition of an equal volume of pooled normal plasma either instantaneously or over two hours of incubation, indicating the presence of an inhibitor. When coagulation factors of the intrinsic pathway were measured, FVIII activity was less than 1% (normal range 60–150%), and coagulation factors IX, XI, and XII were also found to be reduced. Further tests did not detect lupus anticoagulant antibody. The inhibitor for FVIII was quantified using the Bethesda method and showed a high level of 166 BU/mL, but there was no inhibitory activity against factor IX inhibitor (Table 1). Since the patient's coagulation profile had previously been normal, the current results indicated that he had developed acquired haemophilia A (AHA).

## 3. Clinical Course

**Figure 3:**
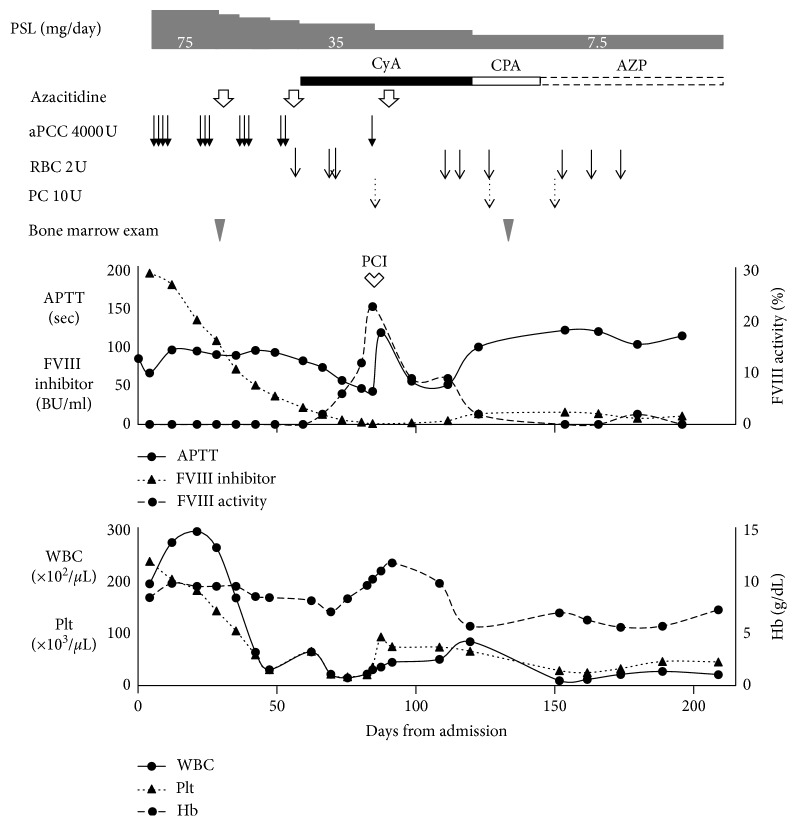
The clinical course of our patient. PSL: prednisolone; CyA: cyclosporine A; CPA: cyclophosphamide; AZP: azathioprine; AZA: azacitidine; aPCC: activated prothrombin complex concentrate; RBC: red blood cells; PC: platelet concentrates; PCI: percutaneous coronary artery intervention.

In order to treat bleeding caused by AHA, we administered activated prothrombin complex concentrate (aPCC; FEIBA, Baxalta©) at a loading dose of 50 U/kg. Immunosuppression with oral prednisolone (PSL) at a dose of 1 mg/kg/day was started on day 3 of hospitalization. Once the patient was less hemorrhagic, further investigations were performed: to assess a possible change in the underlying haematological malignancy, bone marrow examination was undertaken 3 weeks after starting PSL therapy. The bone marrow aspirate revealed normocellular marrow (nucleated cell counts (NCC), 29.7 × 10^4^/*µ*L) and normal myeloid/erythroid (M/E) ratio (1.7). Blast cells accounted for two percentage of NCC. Bone marrow aspirate also shown an elevated number of neutrophils and monocytes with cytoplasmic vacuoles (Table 2) with micromegakaryocyte and erythroblast dysplasia, and the karyotype was determined to be 46, XY, *i*(14) (q10), which was the same abnormality as previously identified (Figures 2(c)–2(e)). Polymerase chain reaction was negative for both *bcr/abl1* fusion gene and *JAK2* mutation. Based on the blood monocytosis and marrow appearances, a diagnosis of chronic myelomonocytic leukaemia (CMML-0) was made. He was not eligible for allogeneic stem cell transplantation because of his age and general status. At the time of bone marrow examination, AHA had not remitted on PSL therapy. FVIII activity was still less than 1%, and inhibitor titer was 109 BU/mL (Figure 3). Platelet count was 142 × 10^9^/L, APTT was 90.9 s, and fibrinogen titer was 131 mg/dL. Assuming a causal relationship between CMML and AHA, we decided to initiate treatment of CMML with the hypomethylating agent, azacitidine (AZA; 75 mg/m^2^ × 7 days, repeated an intervals of 4 weeks), starting 4 weeks after commencing the patient on PSL therapy. After two months of therapy with PSL, the titer of FVIII inhibitor had reduced to 22 BU/mL, but subcutaneous bleeding in his thigh and hemorrhage at the site where blood was drawn had not settled. The laboratory parameter for coagulation did not improve (platelet count, 36 × 10^9^/L; APTT, 94 s; fibrinogen titer, 185 mg/dL; and FVIII activity level, below 3%). We speculated that hypofibrinogenemia was caused by the consumption of fibrinogen on the bleeding with AHA. Cyclosporine A (100 mg/day) was added in to PSL therapy, which improved bleeding events in the extremities and trunk, and FVIII activity level increased to 6% by day 75. However, the patient now began to complain of chest pain and was diagnosed as angina pectoris and was listed for elective percutaneous coronary artery intervention (PCI). On day 87, prior to PCI, the patient received prophylactic platelet transfusion for his low platelet count (25 × 10^9^/L) and aPCC administration as preoperative treatment to prevent bleeding by PCI, but he suffered an acute myocardial infarction. PCI was urgently performed with successful revascularization and resolution of chest pain. He did not receive antiplatelet agent because of his thrombocytopenia. Bleeding symptoms were not evident until 1 month after PCI in which FVIII inhibitor could reach to undetectable level on day 84 and 98 but worsened thereafter coinciding with the appearance of FVIII inhibitor. CyA was switched to 100 mg/day of cyclophosphamide (CPA) with continuous use of PSL on day 117. Prior to the fourth course of AZA therapy, bleeding symptoms emerged and the titer of APTT increased to 100.8 s on day 122, suggesting activation of AHA. Bone marrow reexamination performed on day 138 showed hypocellular marrow, with reduced M/E ratio (1.7 to 0.1). There was no increase in blasts to suggest transformation to acute leukaemic transformation (Table 2). Karyotype analysis, using both G-banding and spectral karyotyping (SKY) methods, revealed an abnormal karyotype, i.e., [idic (14), +1, and der (1; 7)(q10; p10)] (G-banding method [18/20] clone, SKY method [5/5] clone), suggesting clonal evolution (Figure 1(b)) and therefore failure of AZA treatment [6]. AZA was stopped and CPA was replaced with azathioprine 50 mg/day on day 143. He continued on 7.5 mg/day of PSL. He developed recurrent episodes of bruising with APTT index of 100–110 s. Seven months after presenting with AHA, the patient developed gastrointestinal bleeding and died (Figure 3). The response of AHA to treatment was non-CR based on the UK Haemophilia Centre Doctors' Organization criteria [7], despite normalization of FVIII activity and transient disappearance of FVIII inhibitor from hospital day 84 to 98.

## 4. Discussion

The case presented herein had AHA with evolution of MDS-MLD to CMML, which is classified as a rare cause among haematological malignancies. The relationship between MDS or CMML and autoimmune disease has been previously discussed. Approximately 10%–30% MDS or CMML cases are associated with autoimmune diseases [8–10], of which CMML is the most predominant condition [8]. In terms of haematological autoimmune diseases, idiopathic thrombocytopenic purpura, autoimmune hemolytic anaemia, and pure red cell aplasia are commonly seen. Acquired haemophilia complication as a haematological autoimmune disease is rare in MDS or CMML cases. The reason for this may be due to the rare incidental rate of acquired haemophilia. Several cases of AHA complicated with CMML or MDS have been reported [11–16]. Including our case, four out of eight AHA cases went into remission after treatment; three of these cases were administered a hypomethylating agent (AZA or decitabine) for CMML (Table 3). The No. 1 case [11] treated with AZA and panobinostat (histone deacetylase inhibitor) achieved CR. In the three CMML cases (No. 1, 3, and 4) reached to CR of AHA, bone marrow status was examined in only one case (No. 1), which showed CR of CMML status. Bone marrow assessment for the evaluation of CMML status after treatment will be required. In our case, coagulation bypass therapy involving aPCC was performed to stop the bleeding caused by AHA; immunosuppressive therapy by PSL, involving CyA and CPA, was also initiated to prevent the production of AHA antibody. In addition, AZA was administered to treat CMML, which appeared to be the primary disease underlying AHA. During the clinical course of our case, the bleeding symptoms transiently improved after combination therapy with AZA, PSL, and immunosuppressive drugs (CyA, CPA, and azathioprine); however, bleeding tendency recurred in accordance with a reduction of bone marrow cells. We speculated that this bleeding was caused by both AHA-related coagulopathy and thrombocytopenia. Bone marrow examination on day 138 showed the prevalence of abnormal karyotype, i.e., [idic (14), +1, and der (1; 7)(q10; p10)], even if the effect of cell reduction by AZA might remain. The result of additional cytogenetic abnormality implied that the progression of CMML is related to the AHA condition. In non-CR cases, progressive bleeding such as severe subcutaneous and muscle bleeding in case No. 6 [15], intracranial hemorrhage in case No. 2 [11], or gastrointestinal bleeding in case No. 8 (our case) caused the patient's death. These cases had the ineffective condition for haemostatic treatment in the last part of the clinical course.

The mechanism of secondary AHA due to the presence of CMML or MDS remains unclear. In haematological malignancy complicated by AHA, lymphoid malignancy, including lymphoma, chronic lymphoid leukaemia, or macroglobulinaemia, is more common than myeloid malignancy [17]. However, myeloid malignancy is possible to complicate with AIHA infrequently. CMML is predominant in the eight cases shown in Table 3, indicating that CMML may possess a factor responsible for the development of AHA in myeloid malignancy. It is possible that the reason for this is increased inflammatory cytokines levels such as interleukin-6 (IL-6) or tumor necrosis factor alpha that activates the autoimmune function. However, the precise mechanism responsible is unclear. One possibility is that monocytic cells affect autoimmune diseases via the activation of B-cell activating factor (BAFF). In this scenario, BAFF would suppress the production of monocytic cells via a negative feedback mechanism; under normal conditions, BAFF activation would be strictly controlled [18]. Under the CMML conditions, it is possible that increased proportions of monocytic cells, caused by tumorigenesis, do not decrease if BAFF exerts an influence upon the monocytic cell regulation system; the continuation of BAFF activation facilitates the development of autoimmune diseases, such as AHA.

Recombinant factor VIIa or aPCC is generally used for the management of bleeding during surgical treatment of AHA. We chose the use of aPCC because these two agents were used as the similar position for haemostasis, and either choice of these agents would be enough for the management of bleeding. In the present case, elective PCI was scheduled for angina pectoris; the use of aPCC as a pretreatment for the prevention of PCI may have triggered the onset of acute myocardial infarction. With regards to the side effects of aPCC, thrombotic diseases such as disseminated intravascular coagulation, acute myocardial infarction, and pulmonary embolism have been reported [19]. The European Acquired Haemophilia Registry reported that the rate of thrombotic adverse events is 4.8% during aPCC treatment for AHA [20]. Our case had additional risk factors for arteriosclerotic coronary artery disease, such as a smoking habit, hypertension, diabetes mellitus, and obesity. We speculate that the existence of AHA prevented the occurrence of coronary artery disease naturally by inhibiting the intrinsic coagulation pathway and that both normalization of FVIII activity and bypass therapy with aPCC may have stimulated thrombosis of the coronary artery in this patient. A similar case complicated by acute myocardial infarction in partial remission of AHA has been already reported [21]. Consequently, the relationship between the use of haemostatic agents and thrombotic event should be studied further.

Concerning abnormal karyotype, isochromosome 14 (q10) is not common but is recognized in myelocytic malignancy, especially in MDS subtypes RA and CMML [22]. This abnormality can exist as a sole abnormality. Another abnormality, der (1; 7) (q10; p10), is observed in about 1–3% of MDS and less commonly in acute myeloid leukaemia (AML) and myeloproliferative disorders [23]. As chromosome 14 abnormality was observed on three sequential bone marrow tests, chromosome 14 abnormality seems to be the potential cause in this case.

In conclusion, we have presented a case of AHA with CMML. The bleeding symptoms improved transiently on a combination therapy with AZA, PSL, and immunosuppressive drugs. The relative contribution of each therapy cannot accurately be established. Because bleeding tendency becomes steadily worse as CMML status advances, it follows that the control of CMML appears to be essential for the management of AHA.

## Figures and Tables

**Table 1:** Clinical data on admission.

		Normal range
White blood cell	13.6 × 10^9^/L	(3.5–9.7)
Neutrophil	7.6 (56.1%) × 10^9^/L	(38–74%)
Eosinophil	0.16 (1.2%) × 10^9^/L	(0–8.5%)
Basophil	0.03 (0.2%) × 10^9^/L	(0–2.5%)
Monocyte	4.6 (33.6%) × 10^9^/L	(2–10%)
Lymphocyte	1.2 (8.9%) × 10^9^/L	(16–50%)
Red blood cell 312	× 10^4^/*μ*L	(400–550)
Haemoglobin	8.8 g/dL	(13.2–17.2)
Haematocrit	28.1%	(40–52)
MCV	90.1%	(85–103)
Platelet	258 × 10^9^/L	(140–370)
AST	18 IU/L	(13–33)
ALT	12 IU/L	(8–42)
CK	38 IU/L	(62–287)
LD	270 IU/L	(119–229)
Total bilirubin	0.93 mg/dL	(0.2–1.3)
BUN	15.0 mg/dL	(8–20)
Creatinine	0.77 mg/dL	(0.6–1.1)
Total protein	6.4 g/dL	(6.7–8.3)
Albumin	3.7 g/dL	(3.9–4.9)
CRP	2.37 mg/dL	(<0.3)
PT	13.7 s	(10.5–14)
APTT	85.8 s	(24–39)
Fibrinogen	458 mg/dL	(200–400)
FDP	5.6 *μ*g/mL	(<5)
Anticardiolipin *β*_2_GPI	<1.2 U/mL	(<3.5)
Anticardiolipin antibody	<8 U/mL	(<10)
Lupus anticoagulant	1.06 ratio	(<1.3)
Factor VIII activity	<1%	(60–150)
Factor IX activity	34%	(70–130)
Factor XI activity	20%	(75–145)
Factor XII activity	19%	(50–150)
Factor VIII inhibitor	166 BU/mL	(<1)
Factor IX inhibitor	1 BU/mL	(<1)
vWF activity	>200%	(60–170)

**Table 2:** The results of bone marrow aspiration.

	Two years before admission	Hospital day 24	Hospital day 138
NCC (×10^4^/*µ*L)	6.1	29.7	1.2
Megakaryocyte (/*µ*L)	60	156	18
M/E ratio	1.3	1.7	0.1
Erythroid cell (%)	36.8	31.0	71.4
Myeloblast (%)	3.3	2.0	0.8
Promyelocyte (%)	2.9	4.2	0.4
Myelocyte (%)	5.6	8.0	1.0
Metamyelocyte (%)	5.3	4.6	0.2
Stabform cell (%)	8.1	4.8	0.2
Segmented cell (%)	19.8	28.8	6.8
Eosinophilic cell (%)	1.4	0.4	0.8
Basophilic cell (%)	0.2	0.0	0.0
Lymphocyte (%)	9.4	3.0	17.6
Monocyte (%)	6.4	12.0	0.4
Plasma cell (%)	0.2	0.8	0.2

**Table 3:** Cases of acquired haemophilia A with chronic myelomonocytic leukaemia or myelodysplastic syndromes.

No	Age/gender	Disease	Clinical manifestation	APTT (second)	FVIII activity (%)	FVIII inhibitor titer (BU/ml)	Treatment for bleeding and antibody eradication	Anti-tumor agent	Outcome of AHA	Reference
1	54/M	CMML-1	Large ecchymosis at trunk and extremities	73.4	No data	21	PSL	Azacitidine, panobinostat	CR	[11]
2	41/M	CMML-2	Excessive bleeding	91	No data	107.5	CPA, PSL		Non-CR	[11]
3	58/M	CMML-1	Diffuse ecchymosis and spontaneous iliopsoas hematoma	96.3	<1	>200	APCC, PSL, CPA, rituximab, IVIG	Decitabine	CR	[12]
4	71/M	CMML-1	Gross hematuria	118.6	6.7	7.4	HU, rFVIII	Decitabine, CAG regimen	CR	[13]
5	75/F	MDS	Extensive bruising on body and limbs	71	2	420	PSL, pyridoxine		CR	[14]
6	84/M	MDS (RAEB-1)	Subcutaneous and muscle bleeding	71.8	3	3	rFVIIa, PSL		Non-CR	[15]
7	71/M	MDS/MPN	Spontaneous hematoma and muscle hemorrhage	64	24	9	rFVIII, rFVIIa, APCC, PSL, CPA, IVIG		Non-CR	[16]
8	67/M	CMML-0^*∗*^	Subcutaneous and muscle bleeding	85.8	<1	166	APCC, PSL, CyA, CPA, AZP	Azacitidine	Non-CR	Our case

M: male; F: female; CMML: chronic myelomonocytic leukaemia; MDS: myelodysplastic syndromes; PSL: prednisolone; CyA: cyclosporine A; CPA: cyclophosphamide; AZP: azathioprine; HU: hydroxyurea; IVIG: intravenous immunoglobulin; rFVIIa: recombinant factor VIIa; rFVIII: recombinant factor VIII; APCC: activated prothrombin complex concentrate; CR: complete remission; Ref: reference. ^*∗*^Diagnosed by WHO 2016 classification.
